# Probability Forecast Combination via Entropy Regularized Wasserstein Distance

**DOI:** 10.3390/e22090929

**Published:** 2020-08-25

**Authors:** Ryan Cumings-Menon, Minchul Shin

**Affiliations:** 1 The US Census Bureau, 4600 Silver Hill Rd, Suitland-Silver Hill, MD 20746, USA; ryan.r.cumings@gmail.com; 2 Federal Reserve Bank of Philadelphia, Ten Independence Mall, Philadelphia, PA 19106, USA

**Keywords:** entropy regularization, Wasserstein distance, optimal transport, density forecasting, forecast combination, model combination, quantile aggregation

## Abstract

We propose probability and density forecast combination methods that are defined using the entropy regularized Wasserstein distance. First, we provide a theoretical characterization of the combined density forecast based on the regularized Wasserstein distance under the assumption. More specifically, we show that the regularized Wasserstein barycenter between multivariate Gaussian input densities is multivariate Gaussian, and provide a simple way to compute mean and its variance–covariance matrix. Second, we show how this type of regularization can improve the predictive power of the resulting combined density. Third, we provide a method for choosing the tuning parameter that governs the strength of regularization. Lastly, we apply our proposed method to the U.S. inflation rate density forecasting, and illustrate how the entropy regularization can improve the quality of predictive density relative to its unregularized counterpart.

## 1. Introduction

In this paper, we study a class of density forecast combination methods based on a Wasserstein metric. In the univariate case, an equally weighted centroid defined by a Wasserstein metric corresponds to a quantile averaging or vincentized center where quantiles of forecast densities are averaged. The resulting combined density tends to be narrower than the linear opinion rule [1,2,3], which may or not be desirable, depending on the context.

We propose to use the entropy regularized Wasserstein metric to construct a combined density forecast. Like its unregularized counterpart, this combined probability/density can be defined by an optimization problem, but the optimization problem in this case includes an additional regularization term that penalizes densities with low entropy, which ensures the combined density forecast is smooth. One advantage of this approach is that the entropy regularized Wasserstein barycenter can be found in a much more computationally efficient manner than its unregularized counterpart when the input densities are multi-dimensional [4].

While computational efficiency is the most commonly cited reason for using entropy regularization, this paper demonstrates that there is an additional advantage of regularization when it comes to the density combination problem. It provides a way to tune the degree of dispersion of the combined density forecast. To the best of our knowledge, this regularized metric has not been explored in the context of the density forecasting combination problem.

As a part of our discussion, we provide a theoretical characterization of the regularized Wasserstein distance under the Gaussian assumption. More specifically, we show that the regularized Wasserstein barycenter between *two multivariate* Gaussian inputs is multivariate Gaussian. Our proof complements Theorem 1 of [5], which characterizes the regularized Wasserstein barycenter among an *arbitrary number* of *univariate* normal densities. In addition, our result also provides a simple recursive equation that is guaranteed to converge to the variance–covariance matrix.

We proceed as follows. Section 2 formulates a density forecast combination problem with a general metric. Several existing aggregation methods in the literature can be formulated with the choice of a specific metric within this unified framework. After discussing these existing approaches, we introduce our proposal of using the entropy regularized Wasserstein barycenter. Section 3 provides theoretical results that describe the impact of entropy regularization on the combined density under a Gaussian assumption and discusses how this helps improve the quality of the combined density prediction. Section 4 discusses how to set the strength of the entropy regularization in practice and shows that our proposed selection rule achieves a certain notion of optimality. Section 5 provides an empirical exercise that illustrates how entropy regularization improves the quality of density prediction of the U.S. inflation rate relative to the unregularized combined density forecast. Section 6 concludes the article.

## 2. Regularized Wasserstein Barycenter for Density Forecast Combination

This section introduces the density combination problem; see, for example [6]. We assume that agent i∈{1,…,N} at time $t\in N_{+}$ provides a forecast of the density function $p_{it}:R^{d}\to R_{+},$ with distribution function denoted by $P_{it}:R^{d}\to R_{+},$ of the random variable $y_{t+h}$ with $h\in N_{+}$. We are interested in aggregating information contained in the *N* agents’ forecasts to generate a better predictive distribution for $y_{t+h}$.

Throughout the paper, we shall focus on density combinations that can be viewed as a type of average over probability densities. Specifically, those that can be defined as

(1) $${\bar{p}}_{t}=\arg\min_{p_{t}\in P}\sum_{i=1}^{N}D\left(p_{it},p_{t}\right),$$

where $D(p_{i},p_{j})$ is a measure of the discrepancy between the densities $p_{i}$ and $p_{j}.$ When D(·) satisfies the usual properties of a distance metric, which is the case when D(·) is defined as Euclidean or an unregularized Wasserstein metric, then ${\bar{p}}_{t}$ is known as a Fréchet mean, which is a generalization of the average for real numbers. We will refer to ${\bar{p}}_{t}$ as a barycenter to also encompass the more general case in which D(·) is not a metric. As described in Equation (1), we restrict our attention to the case in which ${\bar{p}}_{t}$ is a density forecast with each input density having equal weight, which is known to perform quite well as a combination forecast [7].

A specific choice of metric, $D(p_{i},p_{j})$, will lead to a different combined density, ${\bar{p}}_{t}$. Before introducing our proposed definition of D(·), the entropy regularized Wasserstein metric, the next two sections introduce choices for $D(p_{i},p_{j})$ that lead to well-known density forecast combination methods.

### 2.1. Equal-Weighted Linear Opinion Rule

As a starting point let us consider $D\left(p_{i},p_{j}\right):={\left\|p_{i}-p_{j}\right\|}_{2}^{2}$. Then, Equation (1) becomes

(2) $${\bar{p}}_{t}=\arg\min_{p_{t}\in P}\sum_{i=1}^{N}\int {\left(p_{it}-p_{t}\right)}^{2},$$

which results in the following solution

(3) $${\bar{p}}_{t}=\frac{1}{N}\sum_{i=1}^{N}p_{it}.$$

This can be derived using the first-order condition with respect to $p_{t},$ which is $\sum_{i=1}^{N}\left(p_{it}-{\bar{p}}_{t}\right)=0$.

This solution is known as the linear opinion rule with equal-weighting. This is the prototypical aggregation method both in the forecasting literature and in practice; see, for example [1]. This is a particularly tractable density combination method, as it is equivalent to a mixture density, and it has the additional advantage of being computationally tractable to compute. However, one disadvantage is that it does not preserve the shape of the individual forecast densities. For example, when combining two uni-modal densities, the resulting solution is generally bi-modal.

### 2.2. Quantile Aggregation and the Wasserstein Barycenter

In this section we consider the case in which D(·) is defined as the *p*-Wasserstein metric, which is defined as

(4) $$W_{p}\left(p_{it},p_{jt}\right)={\left(\underset{\varphi \in \Omega (p_{it},p_{jt})}{\inf}\int \|z_{i}-z_{j}{\|}^{p}d\varphi \left(z_{i},z_{j}\right)\right)}^{1/p},$$

where $\Omega (p_{it},p_{jt})$ is the set of all joint distributions $\varphi (z_{i},z_{j})$ that have marginal densities given by $p_{it}$ and $p_{jt}$, respectively. Formally, we write

(5) $$\Omega \left(p_{it},p_{jt}\right)=\left\{\varphi :R^{d}\times R^{d}\to R_{+}^{1}|\forall A\subset R^{d},\;\varphi \left(A,R^{d}\right)=p_{it}\left(A\right)\;\mathrm{and}\;\varphi \left(R^{d},A\right)=p_{jt}\left(A\right)\right\}.$$

In other words, each $\varphi \in \Omega (p_{it},p_{jt})$ is a coupling between the distributions $p_{it}$ and $p_{jt}.$ In the optimal transport literature, the minimizer of (4) is also known as the optimal transport plan. This is because, for any $A,B\subset R^{d},$ φ(A,B) can be interpreted as the amount of mass that is moved from *A* to *B* in order to minimize $E\left({\left\|z_{i}-z_{j}\right\|}_{p}^{p}\right)$ where $z_{i}∼p_{it}$ and $z_{j}∼p_{jt}.$ For more detail on the field of optimal transport, see [8,9].

A special case of this Wasserstein barycenter has a close relation to a recently proposed probability/density forecast combination method in the forecasting literature. More specifically, suppose that input densities are univariate, and ${\bar{p}}_{t}$ is defined as the squared Wasserstein metric, denoted by $D\left(\cdot \right):=W_{2}^{2}\left(\cdot \right);$ in this case, we have

(6) $${\bar{P}}_{t}^{-1}\left(\tau \right)=\frac{1}{N}\sum_{i=1}^{N}P_{it}^{-1}\left(\tau \right),\;\mathrm{for}\;\mathrm{all}\;\;\tau \in \left(0,1\right),$$

where $P_{it}^{-1}\left(\cdot \right)$ and ${\bar{P}}_{t}^{-1}\left(\cdot \right)$ are the quantile function of agent *i* and of the combination method, respectively. This forecast aggregation rule is also known as “quantile aggregation” or “Vincentized distribution” [2,3,10]. We prefer the representation of Equation (1) because this definition can be easily extended to higher dimensional densities or mixed data types (e.g., when some inputs are continuous and others are discrete) unlike quantile aggregation.

The Wasserstein barycenter is known to preserve the shape of input densities, such as log-concavity [11]. For example [12] show that the Wasserstein barycenter of the inputs, $N(\mu_{1},S_{1})$ and $N(\mu_{2},S_{2}),$ is $N(\left(\mu_{1}+\mu_{2}\right)/2,\;S),$ where *S* is the solution of,

(7) $$S={\left(S^{1/2}S_{1}S^{1/2}\right)}^{1/2}/2+{\left(S^{1/2}S_{2}S^{1/2}\right)}^{1/2}/2;$$

see also [13]. This is different than the linear opinion rule, which leads to a mixture of two normal densities with mean $(\mu_{1}+\mu_{2})/2$ and variance $\frac{\sigma_{1}^{2}+\sigma_{2}^{2}}{2}+\frac{{\left(\mu_{1}-\mu_{2}\right)}^{2}}{4}$, which, in contrast, can be expected to be bi-modal whenever $\mu_{1}\neq \mu_{2}$.

Another difference between these two aggregation methods is that the variance of the Wasserstein barycenter is smaller than that of the combined density resulting from a linear opinion rule. This holds for a more general class of input densities as shown in [2] in the univariate case. Of course, a narrow (i.e., sharp) predictive density can be good or bad depending on the underlying distribution of the target variable. It may be desirable to have an ability to flexibly adjust the dispersion of the combined density.

### 2.3. Regularized Wasserstein Barycenter

Now, we turn to our proposal. In this paper, we use a regularized Wasserstein distance [14,15] to combine individual probability forecasts. The regularization term used in this approximation of the Wasserstein metric is given by the negative differential entropy, which, when φ is an absolutely continuous measure, we will define as, $h\left(\varphi \right)=\int_{R^{d}\times R^{d}}\log\left(\frac{d\varphi }{d\lambda }\right)d\varphi ,$ where λ is the Lebesgue measure, and infinity otherwise. We will use h(φ) to define the regularized Wasserstein metric as

(8) $$W_{p,\gamma }\left(p_{it},p_{jt}\right)={\left(\underset{\varphi \in \Omega (p_{it},p_{jt})}{\inf}\int \|z_{i}-z_{j}{\|}^{p}d\varphi \left(z_{i},z_{j}\right)+\gamma h\left(\varphi \right)\right)}^{1/p},$$

where γ>0 controls a strength of regularization. Note that φ is constrained by the same two marginal restrictions as its unregularized counterpart, as described in the definition of $\Omega (p_{it},p_{jt})$. This form of regularization is originally introduced by [14] in order to estimate the Wasserstein metric in a computationally efficient manner using the iterative proportional fitting procedure (IPFP) provided by [16].

When γ=0, there is no regularization, so we have $W_{p,0}\left(p_{it},p_{jt}\right)=W_{p}\left(p_{it},p_{jt}\right).$ One can also show that the optimal coupling, say $\varphi_{\gamma }^{🟉},$ satisfies $\lim_{\gamma \to 0^{+}}\varphi_{\gamma }^{🟉}=\varphi_{0}^{🟉}$ when $\varphi_{0}^{🟉}$ is uniquely defined, and otherwise this limiting value is given by the element of the set of optimal unregularized couplings with maximum entropy [15]. Higher values of γ place more weight on the second term in the objective function, which results in optimal couplings that are smoother and more dispersed than their unregularized counterparts.

Defining $D(p_{it},p_{jt})$ by $W_{2,\gamma }^{2}\left(p_{it},p_{jt}\right)$ results in the combined density

(9) $${\bar{p}}_{t}=\arg\min_{p_{t}\in P}\sum_{i=1}^{N}W_{2,\gamma }^{2}\left(p_{it},p_{jt}\right),$$

which is known as the regularized Wasserstein barycenter. The authors of [4] provided a generalization of the IPFP procedure to find this barycenter that is more computationally efficient than the unregularized case. While computational efficiency is the commonly cited reason for using entropy regularization, as we will see in the later sections, our motivation for regularization is not entirely computational.

For the rest of the paper, we study this regularized Wasserstein barycenter, which is ${\bar{p}}_{t}$ defined in Equation (1) using (8). First, we present analytical results under a parametric assumption that broadens our understanding about the role of the regularization in forecast density combination. Then, we discuss how one can empirically choose the strength of the regularization that would achieve a certain notion of optimality.

## 3. Analytical Results: The Impact of Entropy Regularization

In this section we provide analytical results that describe the impact of entropy regularization on the shape of the barycenter. To better compare this barycenter with its unregularized counterpart in the Gaussian case, as defined above, we will focus on the regularized barycenter when $p_{1}$ and $p_{2}$ are *d*-dimensional multivariate Gaussians (d≥1). The regularized Wasserstein barycenter in this case is defined as

(10) $$\bar{p}\in \arg\min_{q}\left(W_{\gamma }^{2}\left(p_{1},q\right)+W_{\gamma }^{2}\left(p_{2},q\right)\right).$$

The following theorem completely characterizes the resulting barycenter in this case. Like the unregularized case, the theorem shows that regularization does not impact the mean of the barycenter; however, it does have an impact on its variance–covariance matrix.

**Theorem** **1.** *Let $p_{1}$ and $p_{2}$ be Gaussian density functions with means $\mu_{1},\mu_{2}\in R^{d},$ and variance matrices, $S_{1},S_{2}\in R^{d\times d}.$ The regularized Wasserstein barycenter between $p_{1}$ and $p_{2}$ is given by the density function of $N(\mu_{B},S_{B}),$ where $\mu_{B}\in R^{d}$ and $S_{B}\in R^{d\times d}$ are defined by,*

$$\begin{array}{cc} \mu_{B}:= & (\mu_{1}+\mu_{2})/2\\ S_{B}:= & {\left(V/\gamma +I\right)}^{-1}\left(V/2+I\gamma /2+S_{2}\right){\left(V/\gamma +I\right)}^{-1}\\ = & {\left(-V/\gamma +I\right)}^{-1}\left(-V/2+I\gamma /2+S_{1}\right){\left(-V/\gamma +I\right)}^{-1}, \end{array}$$

*where $V\in R^{d\times d}$ is the unique symmetric matrix that satisfies these equalities and −Iγ<V<Iγ.*

*Also, the iterates of the following series converge to V when $V^{\left(0\right)}:=0_{d\times d},$*

$$V^{\left(k+1\right)}=S_{2}-S_{1}+S_{1}{\left(S_{1}+I\gamma /2-V^{\left(k\right)}/2\right)}^{-1}S_{1}-S_{2}{\left(S_{2}+I\gamma /2+V^{\left(k\right)}/2\right)}^{-1}S_{2}.$$

The proof of this result is included in the Appendix A. We prove a slightly more general version of the theorem where the objective function in Equation (10) is a weighted average of $W_{\gamma }^{2}\left(p_{1},q\right)$ and $W_{\gamma }^{2}\left(p_{2},q\right)$. The proof first derives a system of equations that characterizes the barycenter in the case in which the regularized barycenter is Gaussian. Afterward, a fixed point theorem provided by [17] for mappings on partially ordered sets is used to show that this system has a unique solution, and this, along with convexity of Equation (10), implies the regularized barycenter is Gaussian.

Now, we discuss our theoretical results and their implication to the density forecast combination problem.

**Remark** **1.** *(on location). Regularization does not affect the mean of the resulting barycenter, which is a property that may not hold in the more general setting that does not include a normality assumption. For example, suppose the domain of p is [0,1], and $E_{x∼p}\left(x\right)\neq 1/2,$ and consider the barycenter between p and itself. For any fixed density function q, the optimal coupling of the optimization problem that defines $W_{\gamma }^{2}\left(p,q\right)$ converges to $d\varphi \left(z_{1},z_{2}\right)/d\lambda =q\left(z_{1}\right)p\left(z_{2}\right),$ as this is the coupling with maximum entropy that has marginals given by q and p; see for example, [15]. However, the negative entropy of $d\varphi \left(z_{1},z_{2}\right)/d\lambda =p\left(z_{2}\right),$ is less than or equal to that of $d\varphi \left(z_{1},z_{2}\right)/d\lambda =q\left(z_{1}\right)p\left(z_{2}\right),$ for any such fixed density q. We can also ensure these couplings are feasible by defining q to be a uniform density function, so we have $\lim_{\gamma \to \infty }q=1.$ This implies that $\lim_{\gamma \to \infty }E_{x∼q}\left(x\right)=1/2,$ regardless of the $E_{x∼p}\left(x\right).$ Since the unregularized density is given by q=p, and $E_{x∼p}\left(x\right)\neq 1/2,$ the regularization parameter does impact the mean of the barycenter.*

**Remark** **2.** *(on dispersion) Regularization tends to smooth the resulting barycenter, leading to a more dispersed combined density. To understand this point, let us consider a simple example below.*

**Example** **1.** *Consider a case with univariate $p_{it}=N\left(\mu_{it},\sigma^{2}\right)$ and N=2. Then, the original Wasserstein barycenter (quantile averaging) is ${\bar{p}}_{t}=N\left(\left(\mu_{1t}+\mu_{2t}\right)/2,\;\sigma^{2}\right)$. On the other hand the regularized Wasserstein barycenter is ${\bar{p}}_{t}\left(\gamma \right)=N\left(\left(\mu_{1t}+\mu_{2t}\right)/2,\;\sigma^{2}+\gamma /2\right).$*

As this case exemplifies the strength of the regularization controls a dispersion of the combined density. The heavier the regularization the greater dispersed (or, the smoother) density we obtain. This result highlights that the entropy regularization offers an extra flexibility to control the dispersion of the combined density. In the next section, we propose a data-driven way to select the value of γ, the strength of the regularization.

**Remark** **3.** *The normality assumption that we made to obtain the closed-form solution for the barycenter is not needed in practice. The regularized barycenter of probability/density forecasts is well-defined and computationally tractable for a broader context. One can have multiple inputs, non-Gaussian densities, discrete/continuous/mixed distribution. This includes many interesting and empirically relevant situations in economic forecasting such as macroeconomic and financial forecasting. The efficient computation of the regularized Wasserstein distance and barycenter with non-Gaussian input densities is still an active area of research. There is a large literature on computing the regularized barycenter in practice; see for example [4,18,19,20,21,22,23].*

**Remark** **4.** *During the review process for this paper, we became aware of a similar result that was proved independently of ours by [5]. There are two primary differences between these results. First, our result provides the regularized barycenter between two multivariate normal densities, while Theorem 1 in Janati et al. (2020a) provides the barycenter between an arbitrary number of univariate normal densities. Second, our result also provides a recursive formula to compute the variance–covariance matrix of the barycenter, which guarantees a convergence to a desired solution. We appreciate one of referees who pointed out relevant papers.*

*There have also been a number of recent results on a few related barycenters, including those that are modified to avoid the increase in the dispersion of the barycenter caused by regularization using one of the following two techniques. First, a Kullback–Leibler divergence penalty term can be used, with a reference measure given by the product of the input densities, rather than differential entropy. Second, a technique known as debiasing can also be used. For example, the remaining results in [5], as well as the results provided by [24,25], characterize these types of regularized Wasserstein barycenters between Gaussian densities. In contrast to the barycenter we consider, which can be viewed as the original discrete entropy regularized Wasserstein barycenter in the limit as the number of bins diverges, increasing the regularization parameter of these alternative barycenters either decreases or does not change the variance of the barycenter.*

## 4. On Choosing the Strength of the Regularization

This section discusses how to choose the strength of the penalization. Our empirical strategy is to select γ by the value that most accurately fits the observed data. To economize our notation we restrict our discussion to the 1-step-ahead prediction (i.e., h=1). To do so, we regard the regularized barycenter computed at time *t*, ${\bar{p}}_{t}$, as a predictive likelihood for $y_{t+1}$. This predictive likelihood interpretation of the barycenter can be formally justified by the principal-agent framework similar to the one developed by [26]. Suppose we have collected the regularized barycenters and the realized value of the target variable from the initial period (1) to present (t). We write this collection as $I_{t}$. Then, we can define a maximum likelihood estimator for γ at *t* with $I_{t}$ as

(11) $${\hat{\gamma }}_{1:t}^{mle}\in \arg\max_{\gamma \geq 0}\sum_{\tau =1}^{t-1}\log{\bar{p}}_{\tau }\left(y_{\tau +1};\gamma \right),$$

and the combined density prediction for $y_{t+1}$ at time *t* is

(12) $$\hat{p}\left(y_{t+1}|I_{t}\right)={\bar{p}}_{t}\left(y_{t+1};{\hat{\gamma }}_{1:t}^{mle}\right).$$

There is a notion in which this combined density with $\hat{\gamma }$ is optimal. Suppose that $y_{t}{∼}_{i.i.d.}p^{*}\left(y\right)$, and assume that forecasters report a sequence of predictive densities, $p_{i}\left(y\right)$ for $y_{t}$, t=1,2,…,T and i=1,2,…,N. These forecasts are reported before the realization of $y_{t}$, and the barycenter $\bar{p}\left(y;\gamma \right)$ is defined by $p_{i}\left(y\right)$’s and γ>0. Then, the following can be shown under regularity conditions,

$$\frac{1}{T}\sum_{t=1}^{T}\log\bar{p}\left(y_{t};\gamma \right)\to_{p}\int \log\bar{p}\left(y;\gamma \right)p^{*}\left(y\right)dy\;\;\mathrm{as}\;T\to \infty ,$$

for $\gamma \in \Gamma \in R_{+}$. In turn, a maximizer of the left-hand-side term also converges to the maximizer of the right-hand-side term, which is a minimizer of

$$KL\left(\bar{p}\left(y;\gamma \right),p^{*}\left(y\right)\right)=-\int \log\bar{p}\left(y\right)p^{*}\left(y\right)dy+\int \log\left(p^{*}\left(y\right)\right)p^{*}\left(y\right)dy.$$

Therefore, $\hat{\gamma }$ converges to the pseudo-true parameter that minimizes Kullback–Leibler (KL) divergence from the regularized barycenter to the true data generating process. In other words, we find γ that makes the resulting barycenter close to the true data generating process in the limit. This asymptotic thought experiment can be justifiable under quite general conditions, allowing for a range of serial dependence in $y_{t}$ as well as a flexible form of the regularized Wasserstein barycenter implied by $p_{i,t-1}\left(y_{t}\right)$’s. We can operationalize this by recognizing that ${\bar{p}}_{t-1}\left(y;\gamma \right)$ can be viewed as a predictive likelihood for $y_{t}$ formed at time t−1. Then, quasi-MLE theory can be invoked, e.g., [27,28]. We provide a simple example in which the true data generating process follows the autoregressive (AR) process.

**Example** **2.** *Suppose that forecaster 1 and 2 use mean-zero Gaussian AR(1) process to construct their density prediction. The two forecasts differ only by the mean reversion parameter. That is, the means of predictive distribution for forecaster 1 and 2 are $\mu_{1t}=\rho_{1}y_{t-1}$ and $\mu_{2t}=\rho_{2}y_{t-1}$, respectively. Based on our theory in the previous section, the barycenter is ${\bar{p}}_{t-1}\left(y;\gamma \right)=N\left({\bar{\mu }}_{t},\;\sigma^{2}+\gamma /2\right)$ where ${\bar{\mu }}_{t}=\left(\mu_{1t}+\mu_{2t}\right)/2$, and the log density of the regularized barycenter at τ for $y_{\tau +1}$ is*

(13) $$\log\left({\bar{p}}_{\tau }\left(y_{\tau +1};\gamma \right)\right)=-1/2\log\left(2\pi \right)-1/2\log\left(\sigma^{2}+\gamma /2\right)-1/2{\left(\frac{y_{\tau +1}-{\bar{\mu }}_{\tau +1}}{\sqrt{\sigma^{2}+\gamma /2}}\right)}^{2},$$

*and the ML estimator for γ at time t is*

(14) $${\hat{\gamma }}_{1:t}^{mle}\in \arg\max_{\gamma \geq 0}\sum_{\tau =1}^{t-1}\left(-1/2\log\left(2\pi \right)-1/2\log\left(\sigma^{2}+\gamma /2\right)-1/2{\left(\frac{y_{\tau +1}-{\bar{\mu }}_{\tau +1}}{\sqrt{\sigma^{2}+\gamma /2}}\right)}^{2}\right),$$

*which leads to*

(15) $${\hat{\gamma }}_{1:t}^{mle}=2\times \max\left(\frac{1}{\left(t-1\right)}\sum_{\tau =1}^{t-1}{\left(y_{\tau +1}-{\bar{\mu }}_{\tau +1}\right)}^{2}-\sigma^{2},\;0\right).$$

*Now, suppose that the actual data generating process is*

(16) $$y_{t}=\rho_{*}y_{t-1}+v_{t},\;v_{t}{∼}_{i.i.d.}N\left(0,\sigma_{*}^{2}\right).$$

When the simple average of both forecasters’ autoregressive parameter equals $\rho_{*}$, the ML estimate for γ depends on the true conditional variance, $\sigma_{*}^{2}$, and forecasters’ conditional variance. If the sample variance is larger than that of the forecasters, then γ is chosen so that the resulting regularized barycenter has the same variance as the sample variance. On the other hand, if the sample variance is smaller than that of the forecasters, then γ is set to 0. Note that there is an asymmetry in adjusting the variance of the barycenter. This is natural in that the regularization only makes the resulting density smoother. In practice, this may not be a problem if the practitioner’s concern is the combined density being too sharp (e.g., relative to the linear opinion rule).

Note that ${\hat{\gamma }}_{1:t}^{mle}$ converges in probability to $\gamma_{\infty }=2\max\left(\sigma_{*}^{2}-\sigma^{2},0\right)$. The KL divergence between $\bar{p}\left(y_{t+1};\gamma \right)$ and the true conditional density of $y_{t+1}$ at t is minimized at $\gamma =\gamma_{\infty }$. This confirms that our selection rule for γ aims to fit the data well by shaping the regularized barycenter as close as possible to the data generating process.

## 5. Empirical Illustration

In this section, we illustrate our proposed method using macroeconomic data for the U.S. We consider 14 hypothetical forecasters who produce their own 1-step-ahead forecast about the U.S. inflation rate based on the following vector autoregression (VAR) with three variables,

(17) $$Y_{t}=\Phi_{0}+\sum_{i=1}^{4}\Phi_{i}Y_{t-i}+e_{t},\;e_{t}{∼}_{i.i.d}N\left(0,\sum \right),$$

where $Y_{t}$ is a 3×1 vector that consists three quarterly macroeconomic variables, $\Phi_{0}$ is a 3×1 vector, $\Phi_{1},\Phi_{2},\Phi_{3},\Phi_{4},\sum$ are 3×3 matrices. The first two elements of $Y_{t}$ are common to all 14 forecasters: the annualized quarter-over-quarter inflation rate and real GDP growth rate. They differ by the third element of $Y_{t}$. We assign each forecaster a different macroeconomic variable from the FRED-QD database by [29]. A detailed description of the variable used in this exercise is in Table 1.

We compute each forecasters’ 1-step-ahead predictive distribution for the inflation rate at time *t* as $\pi_{t+1|t}∼N\left({\left[\mu_{t+1|t}\right]}_{\left(1,1\right)},{\left[\sum_{t+1|t}\right]}_{\left(1,1\right)}\right)$ where ${\left[x\right]}_{\left(i,j\right)}$ denotes (i,j) element of vector/matrix *x*. These forecasters assume that the 1-step-ahead predictive distribution of $Y_{t+1}$ at *t* is Gaussian, and they use their best guess about the predictive mean and variance to construct the predictive distribution. More specifically, they set these two moments as

(18) $$\mu_{t+1|t}={\hat{\Phi }}_{0,t}+\sum_{p=1}^{4}Y_{t-p+1}^{\prime }{\hat{\Phi }}_{p,t},\;\mathrm{and}\;\sum_{t+1|t}={\hat{\sum }}_{t},$$

where $\left({\hat{\Phi }}_{0,t},{\hat{\Phi }}_{1,t},{\hat{\Phi }}_{2,t},{\hat{\Phi }}_{3,t},{\hat{\Phi }}_{4,t},{\hat{\sum }}_{t}\right)$ is the posterior mean of $p(\Phi_{0},\Phi_{1},\Phi_{2},\Phi_{3},\Phi_{4},\sum |Y_{t:(t-R+1)})$ with a flat prior. We set R=80, meaning that they also use the most recent 20 years of data to construct the predictive distribution.

We let the forecasters to generate their 1-step-ahead predictive distribution for the inflation rate from 2001Q1 to 2018Q4. This leaves us 72 quarters for a forecast evaluation sample. At each point in time, we also combine these 14 predictive densities based on the regularized Wasserstein barycenter with 20 different values of the regularization parameter γ on [0.3,10]. As we explained in the previous section, a larger value of this parameter implies a stronger regularization, and the resulting combined predictive density becomes smoother with a larger variance. We also compute the combined density with γ=0 , which leads to “quantile aggregation” or “Vincentized distribution”. Our computation of the regularized barycenter is based on the algorithm developed and proposed by [19]. The MATLAB toolbox that implements this algorithm is available from https://github.com/gpeyre/2015-SIGGRAPH-convolutional-ot.

**Figure 1:**
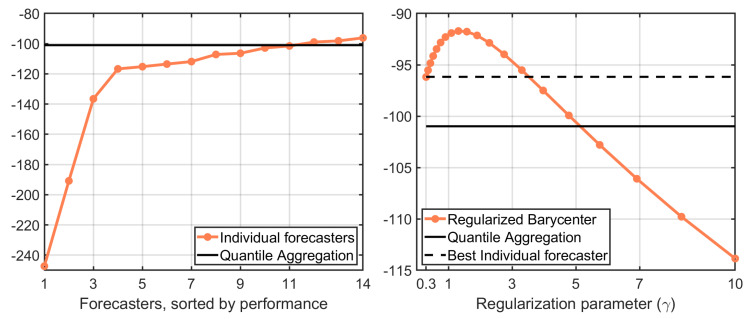
Sum of log predictive score for U.S. inflation rate (2000Q1–2018Q4).

We evaluate each forecaster’s, and other forecast aggregation, methods by the sum of log predictive score, which is a logarithm of the predictive density evaluated at the actualized value, over the evaluation sample. These results are presented in Figure 1. The left panel presents the sum of the log score for individual forecasters sorted by their performance. There is a sizeable difference in their historical performance. The solid line represents the performance based on the quantile aggregation, which aggregates all forecasters in the pool. As found by other research papers, e.g., [2,3] the quantile aggregation method generates a decent predictive distribution, which performs slightly better than the ex-post top 4 forecaster.

The right panel in Figure 1 shows the historical performance of our proposed approach with various choices of regularization parameter, γ. For a wide range of values for γ the regularized barycenter performs better than the quantile aggregation. It does even better than the best individual. This is interesting because we cannot identify the best forecaster a priori.

The optimal value of γ defined in Equation (11) at the end of the evaluation sample would be the value of γ that corresponds to the peak of the curve, which is about ${\hat{\gamma }}_{2018Q4}\approx 1.3$. If we were to use this value at the beginning of the evaluation sample, then the mean difference in the log predictive score between the regularized Wasserstein barycenter and the quantile aggregation would have been 0.12 with the heteroscedasticity and autocorrelation consistent (HAC) standard error being 0.07. This implies that the difference in the peak of the curve and the solid line is statistically significant at 10% confidence level.

To make the γ selection fully adaptive, we also compute the optimal γ sequentially from the beginning to the end of the evaluation sample. That is, we set the predictive density for $y_{t+1}$ as the regularized barycenter with the value of γ that maximizes the objective function defined in Equation (11) *only* using the information available from the beginning of the sample up to *t*. In this way, we do not use any future information when choosing the value of γ. Even in this case the regularized Wasserstein barycenter performs better than the best individual forecaster and the quantile aggregation. The sum of the log predictive score is −93.09, and the mean difference in the log predictive score with the quantile aggregation is 0.11 with the HAC standard error being 0.06. This suggests that the regularized Wasserstein barycenter with the adaptively chosen (e.g., estimated online) γ performs statistically better than its unregularized counterpart, the quantile aggregation, at the 10% significance level. This superior predictive performance of the regularized Wasserstein barycenter relative to the quantile aggregation remains unchanged even when we split the evaluation sample into two. The mean difference in the log predictive score is 0.13 and 0.09 for the first half and the second half of the evaluation sample, respectively.

## 6. Concluding Remarks

This paper proposes to use the entropy regularized Wasserstein barycenter to combine several probability and density forecasts. The entropy regularization smooths the resulting combined forecast, and it offers a flexible way to adjust the dispersion of the predictive density when it is needed. We study the effect of the regularization on the combined density forecast and provide an exact relationship between the strength of the regularization and the variance–covariance matrix of the combined density when input densities are Gaussian. We then provide a way to select the strength of regularization by choosing the regularized barycenter that most closely matches the data. We apply our proposed methodology to the U.S. inflation density forecasting and show how the entropy regularization can improve the quality of the density forecast relative to its unregularized counterpart.

In this article, we restrict weights of each input densities on the final combined density to be pre-determined at some values (i.e., equal weighting). This choice was intentional to focus on studying the role of entropy regularization. In practice, however, it is possible that a subset of input densities might be superior to others, and one may wish to put different weights on each input density. Alternatively, it is desirable to include only a subset of input densities into the combined density and set other weights to zero, see, for example, [30]. For those cases, it is fruitful to develop a data-dependent method that chooses both the regularization strength and those weights simultaneously, which is a topic for future research.

## Figures and Tables

**Table 1:** Variables used in empirical exercises.

$Y^{\left(i\right)}=\left[Y_{1},Y_{2},Y_{3}^{\left(i\right)}\right]$	Used by	Variable Description	FRED-QD Mnemonic
Variable 1 $\left(Y_{1}\right)$	All	Inflation rate	GDPCTPI
Variable 2 $\left(Y_{2}\right)$	All	Real GDP growth rate	GDPC1
Variable 3 $\left(Y_{3}^{\left(i\right)}\right)$	Forecaster 1	Real Personal Consumption Expenditures	PCECC96
	Forecaster 2	Industrial Production Index	INDPRO
	Forecaster 3	All Employees: Total Nonfarm	PAYEMS
	Forecaster 4	Housing Starts: Total Privately Owned Housing Units Started	HOUST
	Forecaster 5	Real Manufacturing and Trade Industries Sales	CMRMTSPLx
	Forecaster 6	Real Crude Oil Prices: West Texas Intermediate (WTI)	OILPRICEx
	Forecaster 7	Real Average Hourly Earnings: Manufacturing	CES3000000008x
	Forecaster 8	10-Year Treasury Constant Maturity Minus 3-Month Treasury Bill	GS10TB3Mx
	Forecaster 9	Real Commercial and Industrial Loans	BUSLOANSx
	Forecaster 10	Real Total Assets of Households and Nonprofit Organizations	TABSHNOx
	Forecaster 11	U.S. / U.K. Foreign Exchange Rate	EXUSUKx
	Forecaster 12	Consumer Sentiment (University of Michigan)	UMCSENTx
	Forecaster 13	S&P’s Common Stock Price Index: Composite	S&P 500
	Forecaster 14	Real Disposable Business Income	CNCFx

Note: All variables are obtained from the FRED-QD database [29]. Inflation rate is computed as a log difference of the GDP deflator (GDPCTPI). Real GDP growth rate is computed as a log difference of the real GDP (GDPC1). All other variables are transformed following [29]. We use the 2019–11 vintage data. Each forecaster constructs a predictive distribution using their own vector autoregression with three variables $Y^{\left(i\right)}=\left[Y_{1},Y_{2},Y_{3}^{\left(i\right)}\right]$ where i=1,2,…,14.

## Appendix A

The authors of [17] provide the following fixed point theorem, which we will use in the proof of Theorem 1.

**Lemma** **A1** **(Ran and Reurings, 2004).**
*Let T be a partially ordered set such that every pair x,y∈T has a lower bound and an upper bound. Furthermore, let d be a metric on T such that (T,d) is a complete metric space. If F:T→T is a continuous, monotone (e.g., either order-preserving or order-reversing) map from T into T such that,*

∃c∈(0,1):d(F(x),F(y))<cd(x,y),∀x>y

*and*

$$\exists \;x_{0}\in T:\;F\left(x_{0}\right)>x_{0}\;or\;F\left(x_{0}\right)>x_{0},$$

*then F has a unique fixed point, $x^{🟉}\in T.$ Also, for all x∈T,*

$$\lim_{n\to \infty }F^{n}\left(x\right)=x^{🟉}.$$

The following result follows from Lemma 1.

**Lemma** **A2.**
*Suppose λ∈(0,1),$T\subset R^{d\times d}$ is the set of symmetric matrices with all eigenvalues in the range $\left(\frac{-\gamma }{2\lambda },\frac{\gamma }{2(1-\lambda )}\right),$ and $S_{1},S_{2}\in R^{d\times d}$ are positive definite matrices. Then there is a unique $V^{🟉}\in T$ such that $F\left(V^{🟉}\right)=V^{🟉},$ where*

$$F\left(V\right):=S_{2}-S_{1}+S_{1}{\left(S_{1}+I\gamma /2-V\left(1-\lambda \right)\right)}^{-1}S_{1}-S_{2}{\left(S_{2}+I\gamma /2+V\lambda \right)}^{-1}S_{2}.$$

*Also, for any V∈T,$\lim_{n\to \infty }F^{n}\left(V\right)=V^{🟉}.$*

**Proof.** Suppose A,B∈T and A>B. First we will establish that F(·) is order-preserving, which is equivalent to F(A)>F(B). Note that,

$$\begin{array}{c} S_{1}\left({\left(S_{1}+I\gamma /2-A\left(1-\lambda \right)\right)}^{-1}-{\left(S_{1}+I\gamma /2-B\left(1-\lambda \right)\right)}^{-1}\right)S_{1}>0\Leftrightarrow \\ {\left(S_{1}+I\gamma /2-A\left(1-\lambda \right)\right)}^{-1}>{\left(S_{1}+I\gamma /2-B\left(1-\lambda \right)\right)}^{-1}\Leftrightarrow \\ -A<-B\Leftrightarrow A>B. \end{array}$$

Similar logic implies that for all such A,B∈T,

$$S_{2}\left({\left(S_{2}+I\gamma /2+B\lambda \right)}^{-1}-{\left(S_{2}+I\gamma /2+A\lambda \right)}^{-1}\right)S_{2}>0\Leftrightarrow A>B,$$

and since F(A)−F(B) is the sum of both of these order-preserving functions, F(·) is also order-preserving. Clearly our bounds on the eigenvalues imply that F(V) is continuous for all V∈T. To show that F is a mapping from *T* into T, note that matrix symmetry is preserved over addition and inversion, so F(V) is symmetric for all V∈T. Also, note that,

$$\begin{array}{c} F\left(-I\gamma /\left(2\lambda \right)\right)=-S_{1}+S_{1}{\left(S_{1}+I\gamma /\left(2\lambda \right)\right)}^{-1}S_{1}>-I\gamma /\left(2\lambda \right)\Leftrightarrow \\ -S_{1}^{1/2}\left(I-{\left(I+S_{1}^{-1}\gamma /\left(2\lambda \right)\right)}^{-1}\right)S_{1}^{1/2}>-I\gamma /\left(2\lambda \right)\Leftrightarrow \\ S_{1}^{1/2}\left(I-{\left(I+S_{1}^{-1}\gamma /\left(2\lambda \right)\right)}^{-1}\right)S_{1}^{1/2}<I\gamma /\left(2\lambda \right)\Leftrightarrow \\ {\left(I+S_{1}2\lambda /\gamma \right)}^{-1}<S_{1}^{-1}\gamma /\left(2\lambda \right)\Leftrightarrow I>0. \end{array}$$

Similar logic can be used to show that F(Iγ/(2(1−λ))<Iγ/(2(1−λ). This also implies the final requirement of Lemma 1. The only remaining requirement of Lemma 2 is the penultimate, which we will establish for A,B∈T such that A>B, and using the norm, d(A,B)=Tr(A−B). Also, let α:={1,−1}, β:={λ−1,λ}, and $∥C∥$ denote the spectral norm of $C\in R^{d\times d}.$ We will use the property $\mathrm{Tr}\left(CD\right)\leq ∥C∥\mathrm{Tr}\left(D\right),$ where $C,D\in R^{d\times d}$ and C,D>0; see for example, [17]. Note that,

$$\begin{array}{c} \mathrm{Tr}(F(A)-F(B))=\\ \sum_{i}\alpha_{i}\mathrm{Tr}\left(S_{i}\left({\left(S_{i}+I\gamma /2+A\beta_{i}\right)}^{-1}-{\left(S_{i}+I\gamma /2+B\beta_{i}\right)}^{-1}\right)S_{i}\right)=\\ \sum_{i}\alpha_{i}\beta_{i}\mathrm{Tr}\left(S_{i}{\left(S_{i}+I\gamma /2+A\beta_{i}\right)}^{-1}\left(B-A\right){\left(S_{i}+I\gamma /2+B\beta_{i}\right)}^{-1}S_{i}\right)=\\ \sum_{i}\alpha_{i}\beta_{i}\mathrm{Tr}\left({\left(S_{i}+I\gamma /2+B\beta_{i}\right)}^{-1}S_{i}S_{i}{\left(S_{i}+I\gamma /2+A\beta_{i}\right)}^{-1}\left(B-A\right)\right)\leq \\ \sum_{i}\alpha_{i}\beta_{i}∥{\left(S_{i}+I\gamma /2+B\beta_{i}\right)}^{-1}S_{i}S_{i}{\left(S_{i}+I\gamma /2+A\beta_{i}\right)}^{-1}∥\mathrm{Tr}\left(B-A\right)<\\ c\mathrm{Tr}\left(B-A\right)\sum_{i}\alpha_{i}\beta_{i}=c\mathrm{Tr}\left(A-B\right), \end{array}$$

where c∈(0,1). The second inequality follows from the matrix $S_{i}{\left(S_{i}+I\gamma /2-A\beta_{i}\right)}^{-1}$ (respectively, $S_{i}{\left(S_{i}+I\gamma /2-B\beta_{i}\right)}^{-1})$ being similar to a symmetric matrix, and with eigenvalues contained in (0,1) because A∈T (B∈T) implies $I\gamma /2-A\beta_{i}>0$ $(I\gamma /2-B\beta_{i}>0).$ □

Next we will establish Theorem 1, which is restated below. This is a slightly more general version of the theorem in the main text where the objective function in Equation (10) is a weighted average of $W_{\gamma }^{2}\left(p_{1},q\right)$ and $W_{\gamma }^{2}\left(p_{2},q\right)$.

**Theorem** **A1.**
*Let λ∈(0,1) and $p_{1}$ and $p_{2}$ be Gaussian density functions with means $\mu_{1},\mu_{2}\in R^{d},$ and variance matrices, $S_{1},S_{2}\in R^{d\times d}.$ The regularized Wasserstein barycenter between $p_{1}$ and $p_{2}$ is given by the density function of $N(\mu_{B},S_{B}),$ where $\mu_{B}\in R^{d}$ and $S_{B}\in R^{d\times d}$ are defined by,*

$$\begin{array}{cc} \mu_{B}:= & \lambda \mu_{1}+\left(1-\lambda \right)\mu_{2}\\ S_{B}:= & {\left(V2\lambda /\gamma +I\right)}^{-1}\left(V\lambda +I\gamma /2+S_{2}\right){\left(V2\lambda /\gamma +I\right)}^{-1}\\ = & {\left(V2(\lambda -1)/\gamma +I\right)}^{-1}\left(V\left(\lambda -1\right)+I\gamma /2+S_{1}\right){\left(V2(\lambda -1)/\gamma +I\right)}^{-1}, \end{array}$$

*where $V\in R^{d\times d}$ is the unique symmetric matrix that satisfies these equalities and −Iγ/(2λ)<V<Iγ/(2(1−λ)).*

*Also, the iterates of the following series converge to V when $V^{\left(0\right)}:=0_{d\times d},$*

$$V^{\left(k+1\right)}=S_{2}-S_{1}+S_{1}{\left(S_{1}+I\gamma /2-V^{\left(k\right)}\left(1-\lambda \right)\right)}^{-1}S_{1}-S_{2}{\left(S_{2}+I\gamma /2+V^{\left(k\right)}\lambda \right)}^{-1}S_{2}.$$

**Proof.** Let $\phi :R^{d}\to R$ be defined as, $\phi \left(z\right):=\exp\left(-{∥z∥}_{2}^{2}/\gamma \right),$ and, for a given function $f:R^{d}\to R,$ we will denote the convolution of f(z) and ϕ(z) as, $f\left(z\right)⊛\phi \left(z\right):=\int_{R^{d}}f\left(t\right)\phi \left(z-t\right)dt.$ When there is little risk of confusion, we will omit the input $z\in R^{d}$ of functions supported on $R^{d}$ in the remainder of the proof. We will characterize the barycenter using the fact that it is the minimizer of the following optimization problem.

(A1) $$\min_{q}\lambda W_{\gamma }^{2}\left(q,p_{1}\right)+\left(1-\lambda \right)W_{\gamma }^{2}\left(q,p_{2}\right).$$

To do so, note that the optimal coupling corresponding to $W_{\gamma }^{2}\left(q,p_{i}\right)$ can be defined by instead solving the dual of (8), which is

(A2) $$\begin{array}{c} w_{i},u_{i}=\arg\max_{w_{i},u_{i}}E_{p_{i}}\left(\log\left(w_{i}\right)\right)+E_{q}\left(\log\left(u_{i}\right)\right)-\gamma \int_{R^{d}\times R^{d}}w_{i}\left(z_{1}\right)u_{i}\left(z_{2}\right)\exp\left(-{∥z_{1}-z_{2}∥}^{2}/\gamma \right)dz_{1}dz_{2}, \end{array}$$

and the optimal coupling can be defined in terms of the dual variables as $d\varphi_{i}\left(z_{1},z_{2}\right)/d\lambda =u_{i}\left(z_{1}\right)\phi \left(z_{1}\right)\phi \left(z_{2}\right)w_{i}\left(z_{2}\right).$ The first order conditions of (A2) are

(A3) $$\begin{array}{cc} p_{i}= & w_{i}\left(u_{i}⊛\phi \right) \end{array}$$

(A4) $$\begin{array}{cc} q= & u_{i}\left(w_{i}⊛\phi \right). \end{array}$$

Also, since the objective function of (A2) is differentiable, an application of the envelope theorem implies

$$\frac{\delta W_{\gamma }^{2}\left(q,p_{i}\right)}{\delta q}=\log\left(u_{i}\right).$$

Thus, the optimum of (A1) can be characterized by the following functional derivative being zero.

$$\begin{array}{cc} \; & \frac{\delta }{\delta q}\left(\lambda W_{\gamma }^{2}\left(q,p_{1}\right)+\left(1-\lambda \right)W_{\gamma }^{2}\left(q,p_{2}\right)\right)=0\Rightarrow \\ \; & \lambda \log\left(u_{1}\right)+\left(1-\lambda \right)\log\left(u_{2}\right)=0 \end{array}$$

After combining this equality with (A3) and (A4), we have that the barycenter can be characterized by the system

$$\begin{array}{c} p_{1}=w_{1}\left(u_{1}⊛\phi_{\gamma /2}\right),p_{2}=w_{2}\left(u_{2}⊛\phi_{\gamma /2}\right)\\ q=u_{1}\left(w_{1}⊛\phi_{\gamma /2}\right)=u_{2}\left(w_{2}⊛\phi_{\gamma /2}\right),\mathrm{and}1=u_{1}^{\lambda }u_{2}^{1-\lambda }. \end{array}$$

This system can be reduced to two equalities after noting that, $p_{i}=w_{i}\left(u_{i}⊛\phi_{\gamma /2}\right)$ and $q=u_{i}\left(w_{i}⊛\phi_{\gamma /2}\right)$ implies

$$q=u_{i}\left(\frac{p_{i}}{u_{i}⊛\phi_{\gamma /2}}⊛\phi_{\gamma /2}\right).$$

After combining both equalities, and noting $u_{1}=u_{2}^{(\lambda -1)/\lambda },$ we have

(A5) $$\begin{array}{c} q=u_{2}^{(\lambda -1)/\lambda }\left(\frac{p_{1}}{u_{2}^{(\lambda -1)/\lambda }⊛\phi_{\gamma /2}}⊛\phi_{\gamma /2}\right)=u_{2}\left(\frac{p_{2}}{u_{2}⊛\phi_{\gamma /2}}⊛\phi_{\gamma /2}\right) \end{array}$$

Let G be defined as the set of functions $g:R^{d}\to R_{+}^{1}$ of the form

$$g\left(z\right)=a\exp(-{\left(z-\mu_{g}\right)}^{⊤}V_{g}^{-1}\left(z-\mu_{g}\right)/2),$$

where $\mu_{g}\in R^{d},$ $V_{g}\in R^{d\times d}$ is a symmetric and invertible matrix, and $a\in R_{++}^{1}.$ It will also be convenient to let $C:G\to R^{d\times d}$ be defined so that $C\left(g\right)=V_{g}$ and $M:G\to R^{d}$ be defined so that $M\left(g\right)=\mu_{g}.$ It is well known that if g,h∈G are Gaussian density functions, then $g^{b},cg,g⊛h,gh\in G,$ where $b,c\in R^{1}$ and b≠0, and it is also straightforward to show

$$\begin{array}{c} C\left(g^{b}\right)=V_{g}/b,C\left(cg\right)=V_{g},\\ C\left(gh\right)={\left(V_{g}^{-1}+V_{h}^{-1}\right)}^{-1},\mathrm{and}C\left(g⊛h\right)=V_{g}+V_{h}. \end{array}$$

Likewise, in the case of M(·), we will also use the properties

$$M\left(g^{b}\right)=\mu_{g},M\left(cg\right)=\mu_{g},M\left(gh\right)=C\left(gh\right)\left(V_{g}^{-1}\mu_{g}+V_{h}^{-1}\mu_{h}\right),\mathrm{and}M\left(g⊛h\right)=\mu_{g}+\mu_{h}.$$

Note that $V_{g}^{-1}+V_{h}^{-1}>0$ is the necessary and sufficient condition for g⊛h to be well defined, and it is straightforward to verify that the properties above also hold over all pairs of g,h∈G when this is the case; for the case of normal density functions, see for example [31]. Next, we will suppose that $u_{2}$ is in G, which, due to (A5), also implies $q,u_{1},w_{1},w_{2}\in G,$ and then show that there exists a unique $u_{2}\in G$ that satisfies (A5). Since (A1) is a strictly convex optimization problem, when a solution to (A1) exists, it can be characterized uniquely by its first-order conditions. Note that, for any pair $u_{i},w_{i}$ that solves (A1), we have that $u_{i}a,w_{i}/a,$ where $a\in R_{++}^{1},$ are also solutions. We avoid complications from this issue by placing the additional restriction on these dual variables that $w_{i}\left(0\right)=1,$ as this ensures strict convexity over this set of dual functions. To see that this is also without loss of generality, note that rescaling the dual variables by $u_{i}a,w_{i}/a$ would not impact the objective function in (A2) because $\int_{R^{d}}q\left(z\right)dz=\int_{R^{d}}p_{i}\left(z\right)dz=1.$ Also, *a* would not impact the first order conditions (A3) and (A4), so it would also not have an impact on *q*. Thus, after providing $u_{2}\in G$ that solves (A5), we will have also shown that this solution is unique even when not restricted to G. Since ϕ, $p_{1},$ and $p_{2}$ are elements of G, and G is closed under multiplication, division, convolution, and exponentiation to the (non-zero) power of (λ−1)/λ, if $u_{2}\in G$ then the functions on both sides of the equality (A5) will also be elements of G. Let $U_{i}:=C\left(u_{i}\right)$ and $\mu_{u}:=M\left(u_{2}\right).$ As noted above, the convolutions in (A5) are only well defined if the following matrix inequalities hold, so we will also require the solution to satisfy these inequalities.

$$I2/\gamma +U_{i}^{-1}>0\mathrm{and}I2/\gamma +U_{i}^{-1}\left(\lambda -1\right)/\lambda >0,$$

which hold if and only if

(A6) $$\begin{array}{c} -2/\gamma I<U_{2}^{-1}<2\lambda /\left(\gamma \left(1-\lambda \right)\right)I. \end{array}$$

It is straightforward to verify that these inequalities are identical to the ones that ensure the optimal coupling is integrable, as this coupling is given by, $d\varphi_{i}\left(z_{1},z_{2}\right)/d\lambda =u_{i}\left(z_{1}\right)\phi \left(z_{1}\right)\phi \left(z_{2}\right)w_{i}\left(z_{2}\right).$ Thus, Fubini’s theorem implies that they are also sufficient conditions for *q* to be integrable. We can find $S_{B}$ by applying C(·) to (A5), which implies

(A7) $$\begin{array}{cc} S_{B}^{-1}= & U_{2}^{-1}+{\left({\left(S_{2}^{-1}-{\left(U_{2}+I\gamma /2\right)}^{-1}\right)}^{-1}+I\gamma /2\right)}^{-1} \end{array}$$

(A8) $$\begin{array}{cc} = & U_{2}^{-1}\left(\lambda -1\right)/\lambda +{\left({\left(S_{1}^{-1}-{\left(U_{2}\lambda /\left(\lambda -1\right)+I\gamma /2\right)}^{-1}\right)}^{-1}+I\gamma /2\right)}^{-1}. \end{array}$$

Let $b_{i}\in \left\{\lambda /\left(\lambda -1\right),1\right\}.$ After three applications of the matrix inversion lemma and simplifying we have that, for each i∈{1,2}

(A9) $$\begin{array}{cc} S_{B}^{-1}-U_{2}^{-1}/b_{i}= & {\left({\left(S_{i}^{-1}-{\left(U_{2}b_{i}+I\gamma /2\right)}^{-1}\right)}^{-1}+I\gamma /2\right)}^{-1}\\ = & {\left({\left(S_{i}^{-1}-I2/\gamma +4/\gamma^{2}{\left(U_{2}^{-1}/b_{i}+I2/\gamma \right)}^{-1}\right)}^{-1}+I\gamma /2\right)}^{-1}\\ = & I2/\gamma -4/\gamma^{2}{\left(S_{i}^{-1}+4/\gamma^{2}{\left(U_{2}^{-1}/b_{i}+I2/\gamma \right)}^{-1}\right)}^{-1}\\ = & I2/\gamma -4/\gamma^{2}S_{i}+4/\gamma^{2}S_{i}{\left(\gamma^{2}/4U_{2}^{-1}/b_{i}+I\gamma /2+S_{i}\right)}^{-1}S_{i}. \end{array}$$

This, along with Equations (A7) and (A8), implies that $U_{2}$ can be characterized by

$$\begin{array}{c} \gamma^{2}/4U_{2}^{-1}-S_{2}+S_{2}{\left(\gamma^{2}/4U_{2}^{-1}+I\gamma /2+S_{2}\right)}^{-1}S_{2}=\\ \gamma^{2}/4U_{2}^{-1}\left(\lambda -1\right)/\lambda -S_{1}+S_{1}{\left(\gamma^{2}/4U_{2}^{-1}\left(\lambda -1\right)/\lambda +I\gamma /2+S_{1}\right)}^{-1}S_{1}. \end{array}$$

After defining *V* as $\gamma^{2}/\left(4\lambda \right)U_{2}^{-1},$ this implies

$$V=S_{2}-S_{1}+S_{1}{\left(S_{1}+I\gamma /2-V\left(1-\lambda \right)\right)}^{-1}S_{1}-S_{2}{\left(S_{2}+I\gamma /2+V\lambda \right)}^{-1}S_{2}.$$

Note that our requirement that $U_{2}^{-1}$ satisfy (A6) can be written in terms of *V* as, −γ/(2λ)I<V<γ/(2(1−λ))I, and Lemma 2 implies that there is a unique solution that satisfies these conditions. The functional form for $S_{B}$ from the statement of this theorem follows from an alternative ordering of the matrix inversion theorem. Specifically, starting from (A9)

$$\begin{array}{cc} S_{B}^{-1}-U_{2}^{-1}/b_{i} & =I2/\gamma -4/\gamma^{2}{\left(S_{i}^{-1}+4/\gamma^{2}{\left(U_{2}^{-1}/b_{i}+I2/\gamma \right)}^{-1}\right)}^{-1}\\ \; & =-U_{2}^{-1}/b_{i}+4/\gamma^{2}\left(\gamma^{2}/4U_{2}^{-1}/b_{i}+I\gamma /2\right){\left(\gamma^{2}/4U_{2}^{-1}/b_{i}+I\gamma /2+S_{i}\right)}^{-1}\\ \; & \times \left(\gamma^{2}/4U_{2}^{-1}/b_{i}+I\gamma /2\right)\\ \; & =-U_{2}^{-1}/b_{i}+\left(2\lambda /(\gamma b_{i})V+I\right){\left(\lambda /b_{i}V+\gamma /2I+S_{i}\right)}^{-1}\left(2\lambda /(\gamma b_{i})V+I\right). \end{array}$$

Thus,

$$\begin{array}{cc} S_{B}^{-1}= & {\left(V2\lambda /\gamma +I\right)}^{-1}\left(S_{2}+V\lambda +I\gamma /2\right){\left(V2\lambda /\gamma +I\right)}^{-1}\\ = & {\left(V2(\lambda -1)/\gamma +I\right)}^{-1}\left(S_{1}+V\left(\lambda -1\right)+I\gamma /2\right){\left(V2(\lambda -1)/\gamma +I\right)}^{-1} \end{array}$$

After applying M(·) to both sides of (A5), we have $M\left(u_{2}^{b_{i}}\left(\frac{p_{i}}{u_{2}^{b_{i}}⊛\phi_{\gamma /2}}⊛\phi_{\gamma /2}\right)\right)=$

(A10) $$S_{B}\left(U_{2}^{-1}\mu_{u}/b_{i}+\left(S_{B}^{-1}-U_{2}^{-1}/b_{i}\right){\left(S_{i}^{-1}-{\left(U_{2}b_{i}+I\gamma /2\right)}^{-1}\right)}^{-1}\left(S_{i}^{-1}\mu_{i}-{\left(U_{2}b_{i}+I\gamma /2\right)}^{-1}\mu_{u}\right)\right).$$

To simplify this expression, we will first establish three intermediate equalities. First, Equations (A7) and (A8) imply

(A11) $$\begin{array}{cc} S_{B}^{-1}-U_{2}^{-1}/b_{i} & ={\left({\left(S_{i}^{-1}-{\left(U_{2}b_{i}+I\gamma /2\right)}^{-1}\right)}^{-1}+I\gamma /2\right)}^{-1}\Rightarrow \\ \; & \left(S_{B}^{-1}-U_{2}^{-1}/b_{i}\right){\left(S_{i}^{-1}-{\left(U_{2}b_{i}+I\gamma /2\right)}^{-1}\right)}^{-1}{\left(U_{2}b_{i}+I\gamma /2\right)}^{-1}\\ \; & ={\left(U_{2}b_{i}+\gamma /2\left(U_{2}b_{i}+I\gamma /2\right)S_{i}^{-1}\right)}^{-1}\\ \; & ={\left(I+\gamma /2\left(I+\gamma /\left(2b_{i}\right)U_{2}^{-1}\right)S_{i}^{-1}\right)}^{-1}U_{2}^{-1}/b_{i}. \end{array}$$

Second, (A11) in turn implies

(A12) $$\begin{array}{cc} \; & \left(S_{B}^{-1}-U_{2}^{-1}/b_{i}\right){\left(S_{i}^{-1}-{\left(U_{2}b_{i}+I\gamma /2\right)}^{-1}\right)}^{-1}S_{i}^{-1}\\ \; & ={\left(I+\gamma /2\left(I+\gamma /\left(2b_{i}\right)U_{2}^{-1}\right)S_{i}^{-1}\right)}^{-1}\left(I+\gamma /\left(2b_{i}\right)U_{2}^{-1}\right)S_{i}^{-1}. \end{array}$$

Third, after an application of the matrix inverse identity to (A7) and (A8)

(A13) $$\begin{array}{cc} S_{B}^{-1} & =U_{2}^{-1}/b_{i}+{\left({\left(S_{i}^{-1}-{\left(U_{2}b_{i}+I\gamma /2\right)}^{-1}\right)}^{-1}+I\gamma /2\right)}^{-1} \end{array}$$

(A14) $$\begin{array}{cc} \; & =U_{2}^{-1}/b_{i}+I2/\gamma -I4/\gamma^{2}{\left(I2/\gamma +S_{i}^{-1}-{\left(U_{2}b_{i}+I\gamma /2\right)}^{-1}\right)}^{-1}, \end{array}$$

which implies

$$\begin{array}{cc} S_{B} & ={\left(U_{2}^{-1}/b_{i}+I2/\gamma -4/\gamma^{2}{\left(\left(U_{2}b_{i}+I\gamma /2\right)\left(I2/\gamma +S_{i}^{-1}\right)-I\right)}^{-1}\left(U_{2}b_{i}+I\gamma /2\right)\right)}^{-1}\\ \; & ={\left(U_{2}^{-1}/b_{i}+I2/\gamma -4/\gamma^{2}{\left(I2/\gamma +\left(I+U_{2}^{-1}\gamma /\left(2b_{i}\right)\right)S_{2}^{-1}\right)}^{-1}U_{2}^{-1}/b_{i}\left(U_{2}b_{i}+I\gamma /2\right)\right)}^{-1}. \end{array}$$

Thus,

(A15) $$\begin{array}{c} S_{B}={\left(U_{2}^{-1}/b_{i}+I2/\gamma \right)}^{-1}{\left(I-{\left(I+\gamma /2\left(I+\gamma /\left(2b_{i}\right)U_{2}^{-1}\right)S_{i}^{-1}\right)}^{-1}\right)}^{-1}. \end{array}$$

We will start with the coefficient on $\mu_{u}$ in (A10). The equalities (A11) and (A15) imply that this term is equal to

$$\begin{array}{cc} S_{B} & \left(U_{2}^{-1}/b_{i}-\left(S_{B}^{-1}-U_{2}^{-1}/b_{i}\right){\left(S_{i}^{-1}-{\left(U_{2}b_{i}+I\gamma /2\right)}^{-1}\right)}^{-1}{\left(U_{2}b_{i}+I\gamma /2\right)}^{-1}\right)\mu_{u}\\ = & {\left(U_{2}^{-1}/b_{i}+I2/\gamma \right)}^{-1}{\left(I-{\left(I+\gamma /2\left(I+\gamma /\left(2b_{i}\right)U_{2}^{-1}\right)S_{i}^{-1}\right)}^{-1}\right)}^{-1}\\ & \times \left(I-{\left(I+\gamma /2\left(I+\gamma /\left(2b_{i}\right)U_{2}^{-1}\right)S_{i}^{-1}\right)}^{-1}\right)U_{2}^{-1}/b_{i}\mu_{u}\\ = & {\left(U_{2}^{-1}/b_{i}+I2/\gamma \right)}^{-1}U_{2}^{-1}/b_{i}\mu_{u}\\ = & {\left(I+U_{2}2b_{i}/\gamma \right)}^{-1}\mu_{u}. \end{array}$$

The equalities (A12) and (A15) imply that the coefficient on $\mu_{i}$ in (A10) can be written as

$$\begin{array}{cc} S_{B} & \left(S_{B}^{-1}-U_{2}^{-1}/b_{i}\right){\left(S_{i}^{-1}-{\left(U_{2}b_{i}+I\gamma /2\right)}^{-1}\right)}^{-1}S_{i}^{-1}\mu_{i}\\ = & {\left(U_{2}^{-1}/b_{i}+I2/\gamma \right)}^{-1}{\left(I-{\left(I+S_{i}^{-1}\gamma /2+U_{2}^{-1}S_{i}^{-1}/b_{i}\gamma^{2}/4\right)}^{-1}\right)}^{-1}\\ & \times {\left(I+\gamma /2\left(I+\gamma /\left(2b_{i}\right)U_{2}^{-1}\right)S_{i}^{-1}\right)}^{-1}\left(I+\gamma /\left(2b_{i}\right)U_{2}^{-1}\right)S_{i}^{-1}\mu_{i}\\ = & {\left(U_{2}^{-1}/b_{i}+I2/\gamma \right)}^{-1}{\left(\gamma /2\left(I+\gamma /\left(2b_{i}\right)U_{2}^{-1}\right)S_{i}^{-1}\right)}^{-1}\left(I+\gamma /\left(2b_{i}\right)U_{2}^{-1}\right)S_{i}^{-1}\mu_{i}\\ = & {\left(U_{2}^{-1}\gamma /\left(2b_{i}\right)+I\right)}^{-1}\mu_{i}. \end{array}$$

After combining these terms, we can define (A10) as the solution to

$$\begin{array}{c} \mu_{q}={\left(I+U_{2}2b_{i}/\gamma \right)}^{-1}\mu_{u}+{\left(U_{2}^{-1}\gamma /\left(2b_{i}\right)+I\right)}^{-1}\mu_{i}\Rightarrow \\ \left(I+U_{2}2b_{i}/\gamma \right)\left(\mu_{q}-{\left(U_{2}^{-1}\gamma /\left(2b_{i}\right)+I\right)}^{-1}\mu_{i}\right)=\mu_{u}\Rightarrow \\ \left(I+U_{2}2b_{1}/\gamma \right)\left(\mu_{q}-{\left(U_{2}^{-1}\gamma /\left(2b_{1}\right)+I\right)}^{-1}\mu_{1}\right)=\left(I+U_{2}2/\gamma \right)\left(\mu_{q}-{\left(U_{2}^{-1}\gamma /2+I\right)}^{-1}\mu_{2}\right). \end{array}$$

Since the matrix inverse identity also implies

$$\begin{array}{c} {\left(U_{2}^{-1}\gamma /\left(2b_{i}\right)+I\right)}^{-1}=I-{\left(U_{2}2b_{i}/\gamma +I\right)}^{-1}, \end{array}$$

we have

$$\begin{array}{cc} \left(I+U_{2}2/\gamma \right)\mu_{q}-U_{2}2/\gamma \mu_{2} & =\left(I+U_{2}2b_{1}/\gamma \right)\mu_{q}-U_{2}2b_{1}/\gamma \mu_{1}\Rightarrow \\ \left(1-b_{1}\right)\mu_{q} & =\mu_{2}-b_{1}\mu_{1}\Rightarrow \\ \left(1+\lambda /\left(1-\lambda \right)\right)\mu_{q} & =\mu_{2}+\lambda /\left(1-\lambda \right)\mu_{1}\Rightarrow \\ \mu_{q} & =\mu_{2}\left(1-\lambda \right)+\lambda \mu_{1}. \end{array}$$

 □
